# The association between acylcarnitine and amino acids profile and metabolic syndrome and its components in Iranian adults: Data from STEPs 2016

**DOI:** 10.3389/fendo.2023.1058952

**Published:** 2023-02-27

**Authors:** Hananeh Taghizadeh, Solaleh Emamgholipour, Shaghayegh Hosseinkhani, Babak Arjmand, Negar Rezaei, Arezou Dilmaghani-Marand, Erfan Ghasemi, Nekoo Panahi, Hojat Dehghanbanadaki, Robabeh Ghodssi-Ghassemabadi, Niloufar Najjar, Mojgan Asadi, Mohsen khoshniat, Bagher Larijani, Farideh Razi

**Affiliations:** ^1^ Endocrinology and Metabolism Research Center, Endocrinology and Metabolism Clinical Sciences Institute, Tehran University of Medical Sciences, Tehran, Iran; ^2^ Department of Clinical Biochemistry, School of Medicine, Tehran University of Medical Sciences, Tehran, Iran; ^3^ Diabetes Research Center, Endocrinology and Metabolism Clinical Sciences Institute, Tehran University of Medical Sciences, Tehran, Iran; ^4^ Cell Therapy and Regenerative Medicine Research Center, Endocrinology and Metabolism Molecular-Cellular Sciences Institute, Tehran, Iran; ^5^ Non-Communicable Diseases Research Center, Endocrinology and Metabolism Population Sciences Institute, Tehran University of Medical Sciences, Tehran, Iran; ^6^ Metabolic Disorders Research Center, Endocrinology and Metabolism Molecular-Cellular Sciences Institute, Tehran University of Medical Sciences, Tehran, Iran; ^7^ Department of Biostatistics, School of Medical Sciences, Tarbiat Modares University, Tehran, Iran; ^8^ Metabolomics and Genomics Research Center, Endocrinology and Metabolism Molecular-Cellular Sciences Institute, Tehran University of Medical Sciences, Tehran, Iran; ^9^ Osteoporosis Research Center, Endocrinology and Metabolism Clinical Sciences Institute, Tehran University of Medical Sciences, Tehran, Iran

**Keywords:** amino acid, acylcarnitine, metabolic syndrome, metabolomics, metabolites

## Abstract

**Background:**

Evidence, albeit with conflicting results, has suggested that cardiometabolic risk factors, including obesity, type 2 diabetes (T2D), dyslipidemia, and hypertension, are highly associated with changes in metabolic signature, especially plasma amino acids and acylcarnitines levels. Here, we aimed to evaluate the association of circulating levels of amino acids and acylcarnitines with metabolic syndrome (MetS) and its components in Iranian adults.

**Methods:**

This cross-sectional study was performed on 1192 participants from the large–scale cross-sectional study of Surveillance of Risk Factors of non-communicable diseases (NCDs) in Iran (STEP 2016). The circulating levels of amino acids and acylcarnitines were measured using liquid chromatography-tandem mass spectrometry (LC-MS/MS) in individuals with MetS (n=529) and without MetS (n=663).

**Results:**

The higher plasma levels of branched-chain amino acids (Val, Leu), aromatic amino acids (Phe, Tyr), Pro, Ala, Glu, and the ratio of Asp to Asn were significantly associated with MetS, whereas lower circulating levels of Gly, Ser, His, Asn, and citrulline were significantly associated with MetS. As for plasma levels of free carnitine and acylcarnitines, higher levels of short-chain acylcarnitines (C2, C3, C4DC), free carnitine (C0), and long-chain acylcarnitines (C16, C18OH) were significantly associated with MetS. Principal component analysis (PCA) showed that factor 3 (Tyr, Leu, Val, Met, Trp, Phe, Thr) [OR:1.165, 95% CI: 1.121-1.210, P<0.001], factor 7 (C0, C3, C4) [OR:1.257, 95% CI: 1.150-1.374, P<0.001], factor 8 (Gly, Ser) [OR:0.718, 95% CI: 0.651-0.793, P< 0.001], factor 9 (Ala, Pro, C4DC) [OR:1.883, 95% CI: 1.669-2.124, P<0.001], factor 10 (Glu, Asp, C18:2OH) [OR:1.132, 95% CI: 1.032-1.242, P= 0.009], factor 11 (citrulline, ornithine) [OR:0.862, 95% CI: 0.778-0.955, P= 0.004] and 13 (C18OH, C18:1 OH) [OR: 1.242, 95% CI: 1.042-1.480, P= 0.016] were independently correlated with metabolic syndrome.

**Conclusion:**

Change in amino acid, and acylcarnitines profiles were seen in patients with MetS. Moreover, the alteration in the circulating levels of amino acids and acylcarnitines is along with an increase in MetS component number. It also seems that amino acid and acylcarnitines profiles can provide valuable information on evaluating and monitoring MetS risk. However, further studies are needed to establish this concept.

## Introduction

Metabolic syndrome (MetS) is a collection of interrelated cardiometabolic abnormalities, including central adiposity, hyperglycemia, hypertension, hypertriglyceridemia, and low level of high-density lipoprotein cholesterol (HDL-C). These features are strongly linked to the development of type 2 diabetes (T2D) and cardiovascular disease and increased mortality (1). This condition is a global epidemic disorder affecting about 20-25% of adults worldwide (2). When compared across regions, it was estimated that 32-47.6% of the Iranian population was afflicted with MetS (3).

Recently, tremendous efforts have been devoted to addressing the MetS pathogenesis; however, there is considerable uncertainty in this regard. Additionally, there is no optimal screening tool and treatment for this disease, necessitating the identification of novel target-based approaches for better diagnostic and treatment modalities (1). Currently, the clinical relevance of metabolomics to identify a collection of biomarkers for the detection, prediction, and monitoring of MetS and its associated metabolic abnormalities comes into the center of interest (4–6).

Various studies albeit with conflicting results have suggested that cardiometabolic risk factors, including obesity, T2D, dyslipidemia, and, hypertension are highly associated with changes in metabolic signature, especially plasma amino acids and acylcarnitines levels (7–13).

The amino acid availability has been implicated in regulating intracellular signaling, hormonal secretion, and energy homeostasis. For example, the branched-chain amino acids (BCAAs; leucine, isoleucine, valine) play important roles in the regulation of insulin secretion, glucose, lipid metabolism, central nervous system control of food intake, and energy balance (14, 15).

Current evidence reports elevations in the profile of branched-chain amino acids (BCAAs; leucine, isoleucine, valine) and aromatic amino acids (AAAs; phenylalanine, tyrosine) in individuals with type 2 diabetes(T2D), insulin resistance, and obesity (4). Moreover, BCAA, Phenylalanine (Phe), and tyrosine (Tyr) could predict the risk of T2D, MetS, and cardiovascular disorders before disease manifestation (16–18).

The change in acylcarnitine profile could reflect dysregulation of fatty acid metabolism and mitochondrial function and point toward the presence of fatty acid oxidation and organic acid metabolism disorders (10, 19). Change in circulating levels of acylcarnitines has also been reported in patients with MetS (7, 20). Additionally, alteration of acylcarnitine plasma levels, including tetradecenoylcarnitine (C14:1), tetradecadienylcarnitine (C14:2), octadecenoylcarnitine (C18:1), and malonylcarnitine/hydroxy butyryl carnitine (C3DC+C4OH) are linked to both T2D and prediabetes conditions (9).

There is growing attention to the analysis of metabolite profiles in the context of metabolic disorders (4, 21). However, several studies, albeit with conflicting results, showed a change in the pattern of amino acids and acylcarnitine profile in patients with MetS. Moreover, the criteria used for MetS definitions and studied populations vary. Despite studies in other countries and ethnicities, no data has been reported to evaluate a correlation of amino acid and acylcarnitine profile with MetS in Iranian adults. Hence, we sought to assess the association of circulating levels of amino acids and acylcarnitines with MetS and its components in an attempt to identify candidate biomarkers for the risk of MetS.

## Participants and methods

### Study design and participants

This study was performed on participants (n= 1192) randomly selected from the large–scale cross-sectional study of Surveillance of Risk Factors of non-communicable diseases (NCDs) in Iran (STEP 2016), which has been well discussed elsewhere (22). In brief, through a systematic cluster random sampling, proportional to the adult population of each province, STEP16 was planned to collect data on 31,050 Iranian subjects (3,105 clusters) aged ≥18 years living in urban and rural areas of 31 provinces of Iran in 2016.

All participants underwent measurement of anthropometric indices including height, weight, Body Mass Index (BMI), waist circumference (WC), hip circumference (HC), and waist-to-hip ratio (WHR) by standard protocols which are consistent with WHO protocols. The systolic blood pressure (SBP) and diastolic blood pressure (DBP) were measured three times, and the average value of the second and third readings was used for analysis. After overnight fasting, blood samples were collected, and plasma was separated by centrifugation at 4°C. A portion of plasma was used for the measurement of clinical parameters including fasting plasma glucose (FPG), triglyceride (TG), high-density lipoprotein-cholesterol (HDL-C), and total cholesterol, and another portion was stored at -70°ϲ until metabolomics analysis. Plasma levels of FPG, TG, and HDL-C were measured based on the standard procedure using the auto analyzer (Cobas C311, Roche Diagnostics).

According to the National Cholesterol Education Programme Adult Treatment Panel III (NCEP ATP III) definition (23), participants were classified into two groups; the MetS group and the non-MetS group. Participants were defined as having MetS if they had at least three of the following five items: 1) Waist circumference ≥ 102 cm in men and 88 cm in women; 2) FPG ≥100 mg/dl (or diagnosed diabetes); 3) TGs ≥150 mg/dl; 4) HDL-C <40 mg/dl in men and <50 mg/dl in women; and 5) SBP ≥130 mmHg and DBP ≥85 mmHg.

Ethical approval for the current study was obtained from the ethics committee of the Endocrine and metabolism research institute (IR.TUMS.EMRI.REC. 1395.00141). It should be noted that the objectives and study protocol were described for all eligible individuals and written informed consent was obtained from each participant before the study.

### Metabolomics analysis

Metabolites including 20 amino acids and 30 acylcarnitines were analyzed in the fasting plasma of participants by targeted approach. The full details of method development, validation, and preparation protocol have been completely described elsewhere (24–26). Briefly, samples were mixed with isotope-labeled internal standards and derivatized with butanoic-HCL. Instrumental analysis was conducted on Thermo Scientific Ultimate 3000 liquid chromatography system coupled with an AB SCIEX API 3200 Triple Quadrupole mass spectrometer with ESI positive ion mode. Finally, the data was processed using Multiquant 3.0.2 software.

### Statistical analysis

For comparison of the metabolite concentrations between the MetS group and the control one. Student’s t-test and Mann-Whitney were used as appropriate.

For the identification of metabolites statistically associated with MetS, binary logistics regression was used, where fitness for the model was checked by the Hosmer–Lemeshow goodness of fit model. A 95% confidence interval and a value of less than 0.05 were used as statistically significant levels.

Factor (principal component) analysis was performed to determine the potential pattern of metabolites associated with MetS. Kaiser-Meyer-Olkin (KMO) and Bartlett sphericity tests were used to evaluate the suitability for factor analysis. Varimax rotation was applied to facilitate their interpretation. According to the scree plot, only the components with eigenvalues greater than 1 were kept. Metabolites with a factor loading ≥0.4 were considered important for further analysis.

Statistical analysis was conducted using SPSS 26 (IBM Corp., Armonk, NY, USA) and Graph Pad Prism 9 (GraphPad Software, San. Diego, CA). There was no outlier deletion. Data were z-transformed and analyzed. The Benjamini-Hochberg false discovery rate (FDR) was calculated to adjust the P-value for multiple comparisons. A P-value less than 0.05 was considered statistically significant.

## Results

### Baseline characteristics of study population

A total of 1192 participants were enrolled in this study: 529 patients with MetS (202 men and 327 women) and 663 ones without MetS (368 men and 295 women). The demographic, anthropometric, and biochemical characteristics of all participants are summarized in **Table 1**. The mean age for MetS and non-MetS groups was 56.75 ± 11.48 and 55.69 ± 12.45 years, respectively. There was not any significant difference between patients and controls in terms of years of education. As expected, individuals with MetS had higher BMI, waist circumference, hip circumference, and a worse metabolic profile reflected by higher levels of FPG, TG, and total cholesterol, while lower levels of HDL-C were in comparison with the subjects without MetS.

**Table 1:** The clinical characteristics and biochemical indices between the MetS group and non-MetS group.

N	Non-MetS group	MetS group	P-value
	663	529	
Gender, n (%)			0.000
Women	295 (44.49)	327 (61.81)	
Men	368 (55.51)	202 (38.19)	
Education (year)			0.281
<1 year	168 (25.34)	147 (27.79)	
1-7 year	221 (33.33)	189 (35.73)	
8-12 year	186 (28.05)	139 (26.28)	
>12 year	88 (13.27)	54 (10.21)	
Age (year)	55.69 ± 12.45	56.75 ± 11.48	0.133
BMI (kg/m²)	25.75 ± 4.61	30.16 ± 4.75	<0.001
SBP (mmHg)	126.73 ± 19.78	138.65 ± 20.27	<0.001
DBP (mmHg)	77.67 ± 10.88	83.88 ± 12.14	<0.001
FPG (mg/dL)	93.97 ± 27.29	115.18 ± 44.30	<0.001
TG (mg/dL)	104.34 ± 57.23	176.82 ± 121.96	<0.001
HDL-C (mg/dL)	45.38 ± 11.76	36.02 ± 8.94	<0.001
Cholesterol (mg/dL)	164.38 ± 34.67	172.05 ± 37.38	<0.001
WC (cm)	89.79 ± 13.05	101.27 ± 10.42	<0.001
HC (cm)	98.83 ± 11.17	106.78 ± 9.79	<0.001
Smoking	125 (18.85)	47(8.88)	<0.001

BMI, body mass index; TG, triglycerides; FPG, fasting plasma glucose; LDL-C, low-density lipoprotein cholesterol; HDL-C, high-density lipoprotein cholesterol; WC, waist circumference; HC, hip circumference; SBP, systolic blood pressure; DBP, diastolic blood pressure; Mets, metabolic syndrome. Results are represented as mean ± standard deviation (SD) for continuous variables and n (percentages, %) for categorical variables.

### Altered plasma amino acid and acylcarnitine levels in MetS

Binary logistic regression analysis of 50 metabolites; 20 amino acids and 30 acylcarnitines has been demonstrated in **Supplementary Table 1**. The higher plasma levels of branched-chain amino acids (Val, Leu), aromatic amino acids (Phe, Tyr), Pro, Ala, Glu, and the ratio of Asp to Asn were significantly associated with MetS, whereas lower circulating levels of Gly, Ser, His, Asn, and citrulline were significantly associated with MetS.

After age and sex adjustment, all the above-mentioned amino acids plus Met, Trp, and the ratio of Asp to Asn showed a statistically significant association with MetS.

As for plasma levels of free carnitine (C0) and acylcarnitines, higher levels of C0, short-chain acylcarnitines (C2, C3, C4DC), and long-chain acylcarnitines (C16, C18OH) were significantly associated with MetS. However, the lower plasma level of C4 was significantly lower in the MetS group than in the non-MetS group. Multivariate analysis showed that circulating levels of C0 and acylcarnitines including C3, C4DC, C5, C16, and C18OH were associated with MetS independent of age and sex as covariates.

The univariate analysis of 50 metabolites; 20 amino acids and 30 acylcarnitines between the MetS group and non-MetS group has been demonstrated in **Supplementary Table 2**. Our data showed that plasma levels of C0, short-chain acylcarnitines (C0, C2, C3, C4DC), and long-chain acylcarnitines (C16, C18OH) were significantly higher in patients with MetS in comparison to those in the non-MetS group. However, the plasma level of C4 was significantly lower in the MetS group than in the non-MetS group.

The Plasma levels of branched-chain amino acids (Val, Leu), aromatic amino acids (Phe, Tyr), Pro, Ala, Glu, and the ratio of Asn to Asp were all significantly higher in the MetS group compared to those in the non-MetS group. However, circulating levels of Gly, Ser, His, Asn, and citrulline were significantly lower in the MetS group compared to those in the non-MetS group.

Given the statistically significant difference in the number of men and women in the two study groups, possible changes in the plasma level of amino acids and acylcarnitines were also calculated depending on gender (**Supplementary Table 3**).

According to sex differences, the plasma level of C0, short-chain acylcarnitines (C3, C3DC, C4DC, C5, C5OH, and C5DC), medium-chain acylcarnitines (C8, C10, C10:1, and C12) and long-chain acylcarnitines (C14, C14:2, C14 OH, C16, C16 OH, C16:1OH, and C18) were significantly higher in men in comparison to those in women in MetS group. A similar pattern was also found when comparing men and women in the non-MetS group.

As for amino acids, except for Ala, Arg, Thr, Ser, His, and Lys all amino acids were significantly higher in men in comparison to those in women in both groups. However, Arg plasma level was significantly higher in men in comparison to those in women in the MetS group.

### Altered circulating levels of amino acid and acylcarnitine profile with MetS components

The association of 19 amino acids and acylcarnitines (that were shown to be significantly different in MetS and non- MetS groups) with MetS components was shown in **Table 2**. All participants were categorized into four groups based on the number of MetS components (increased waist circumference, hypertension, hyperglycemia, hypertriglyceridemia, and decreased HDL-C concentration). These groups include ones who didn’t have any MetS component (0-component group), ones having only one MetS component (1-component group), ones with two MetS components (2-component group), and ones who had between 3 and 5 MetS components (3-5-component group).

**Table 2:** The levels of amino acids and acylcarnitines in different groups according to the number of metabolic syndrome components.

Metabolites (µmol/L)	0 component	1 component	2 components	3-5 components
	n= 152	n= 228	n= 283	n= 529
C0	52.742 ± 1.019	55.975 ± 0.824**	55.711 ± 0.762**	58.121 ± 0.577***
C2	14.585 ± 0.432	14.552 ± 0.349	13.999 ± 0.232	14.872 ± 0.187
C3	0.732 ± 0.021	0.845 ± 0.022***	0.875 ± 0.022***	0.940 ± 0.018***
C4	0.488 ± 0.041	0.543 ± 0.037	0.504 ± 0.033	0.510 ± 0.019**
C4DC	0.064 ± 0.002	0.065 ± 0.002	0.071 ± 0.002	0.080 ± 0.002***
C16	0.184 ± 0.005	0.180 ± 0.003	0.183 ± 0.004	0.190 ± 0.002
C18OH	0.009 ± 0.000	0.009 ± 0.000	0.008 ± 0.000	0.010 ± 0.000
Alanine	360.929 ± 6.661	385.890 ± 6.327**	404.722 ± 5.154***	452.150 ± 4.135***
Glutamic Acid	66.334 ± 1.803	65.979 ± 0.827	67.902 ± 0.822*	69.274 ± 0.584***
Leucine	116.537 ± 1.790	117.724 ± 1.625	125.256 ± 1.676**	131.710 ± 1.169***
Phenylalanine	61.281 ± 0.911	63.150 ± 0.803	64.408 ± 0.667**	66.369 ± 0.643***
Tyrosine	65.275 ± 1.034	68.478 ± 0.863**	70.986 ± 0.875***	74.753 ± 0.653***
Valine	237.840 ± 3.288	242.690 ± 3.053	262.019 ± 3.171***	277.448 ± 2.321***
Citrulline	41.686 ± 0.818	40.265 ± 0.688	38.520 ± 0.632**	36.930 ± 0.437***
Glycine	278.421 ± 5.993	289.125 ± 5.625	262.474 ± 4.836**	257.256 ± 3.335***
Proline	233.227 ± 5.735	241.445 ± 5.529	256.642 ± 5.167**	264.772 ± 3.665***
Serine	111.587 ± 2.328	110.675 ± 2.047	103.309 ± 1.660**	97.003 ± 1.196***
Histidine	84.677 ± 1.227	84.979 ± 1.121	84.378 ± 0.959	81.901 ± 0.777
Asparagine	52.099 ± 1.627	50.258 ± 1.322	47.591 ± 1.208*	44.490 ± 0.790***

The number of metabolic syndromes components was determined according to the National Cholesterol Education Programme Adult Treatment Panel III (NCEP ATP III) definition (23).

Results are represented as mean ± standard error of the mean (SEM).

*P-value< 0.05.

**P-value< 0.01.

***P-value< 0.001.

Variables with the statistical difference between the metabolic syndrome group and without one were evaluated for the analysis of the association of amino acids and acylcarnitines with metabolic syndrome components.

Comparisons among the four groups were done by one-way ANOVA.

When significant differences were found, the Tukey post hoc test was used for multiple comparisons.

We found an increasing trend regarding plasma levels of amino acids; Ala, Tyr, free carnitine, and acylcarnitines; C3 as the number of components increased. The 2-component group and 3-5- component group had a higher level of Glu, Leu, Phe, Val, Citrulline, Gly, Pro, Ser, and Asn in comparison with the 0-component group. The other acylcarnitines and amino acids were not associated with MetS components.

### Amino acid profiles extracted by PCA were associated with MetS and its components

**Figure 1:**
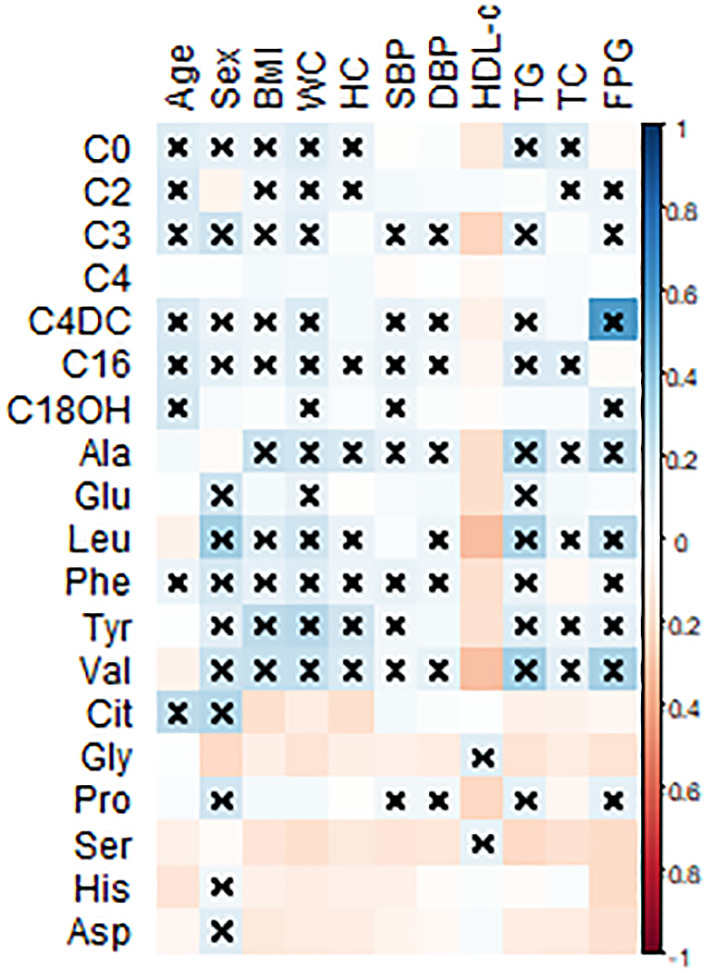
Pearson’s correlation coefficients were calculated for the 12 amino acids and 7 acylcarnitines with metabolic-related variables. BMI, body mass index; WC, waist circumference; HC, hip circumference, FPG, fasting plasma glucose; SBP, systolic blood pressure; DBP, diastolic blood pressure; TC, total cholesterol; TG, triglyceride; HDL-c, high-density lipoprotein-cholesterol; Leu, leucine; Val, valine; Tyr, tyrosine; Phe, phenylalanine; Glu, glutamic acid; Ala, alanine; Gly, glycine; Cit, citrulline, His, histidine; Asn, asparagine; Pro, proline. All statistically significant associations were marked with a multiplication sign. Statistical differences are shown as ^×^P < 0.05.

Spearman’s correlation coefficient analysis was done to evaluate the correlation of the 12 amino acids and 7 acylcarnitines with the metabolic characteristics linked to MetS (**Figure 1**). As shown in **Figure 1**, plasma levels of Leu, Val, and Phe have a moderate but significant correlation with FPG and TG levels. Moreover, C4DC was significantly associated with FPG levels.

Kaiser-Meyer-Olkin (KMO) test was used to assess the suitability of our data for Factor Analysis. Here, the KMO value was 0.858 which indicates the sampling is adequate. Moreover, Bartlett’s test of Sphericity was significant (P-value < 0.001). Factors 1-13 had eigenvalues of more than 1 and explained 73.38% of the cumulative variance that has been depicted by using a scree plot (**Supplementary Figure 1**). So, we extracted 13 factors through Principal Component Analysis (PCA) and the loadings after varimax rotation and Kaiser Normalization were listed in **Supplementary Table 4**. Only metabolites with loading ≥ 0.4 are included in the factors (**Table 3)**.

**Table 3:** Extracted factors based on PCA and loadings.

PC1	Loading	PC3	Loading	PC6	Loading	PC11	Loading
C16:1OH	0.825	Tyr	0.799	C4OH	0.694	Cit	0.787
C14	0.819	Leu	0.776	C8:1	0.688	Orn	0.659
C16	0.774	Val	0.758	C2	0.641	PC12	Loading
C18:1	0.751	Met	0.740	PC7	Loading	Arg	0.709
C18	0.733	Trp	0.712	C0	0.698	PC13	Loading
C14OH	0.708	Phe	0.616	C3	0.675	C18:1OH	0.827
C16OH	0.680	Thr	0.429	C4	0.527	C18OH	0.599
C12	0.591	PC4	Loading	PC8	Loading		
C16:1	0.679	Lys	0.906	Gly	0.815		
C14:1	0.653	Glu	0.894	Ser	0.667		
C14:2	0.424	Asp	0.711	PC9	Loading		
PC2	Loading	His	0.683	Ala	0.624		
C8	0.946	PC5	Loading	Pro	0.565		
C10	0.939	C5DC	0.450	C4DC	0.468		
C10:1	0.919	C5:1	0.873	PC10	Loading		
C6	0.801	C5OH	0.762	Glu	0.726		
		C3DC	0.577	C18:2OH	0.555		
		C5	0.559	Asp	0.549		

Lys, lysine; Leu, leucine; Val, valine; Met, methionine; Trp, tryptophane; Tyr, tyrosine; Phe, phenylalanine; Thr, threonine; Glu, glutamic acid; Ala, alanine; Gly, glycine; Cit, citrulline, His, histidine; Asn, asparagine; Pro, proline; Serine, Ser;serine; Asp, aspartic acid; Orn, ornithine; Arg, arginine.

The results of age and sex-adjusted odds ratio (OR) for the association between metabolites with VIP score values above 1.0 and metabolic syndrome (MetS) were shown in **Table 4**. After adjustment for age and sex, factor 3 (Tyr, Leu, Val, Met, Trp, Phe, Thr), factor 7 (C0, C3, C4), factor 8 (Gly, Ser), factor 9 (Ala, Pro, C4DC), factor 10 (Glu, Asp, C18:2OH), factor 11 (citrulline, ornithine) and 13 (C18OH, C18:1 OH) were independently correlated with metabolic syndrome. Factor 3 (OR:1.165, 95% CI: 1.121-1.210, P<0.001), 7 (OR:1.257, 95% CI: 1.150-1.374, P<0.001), 9 (OR:1.883, 95% CI: 1.669-2.124, P<0.001), 10 (OR:1.132, 95% CI: 1.032-1.242, P= 0.009) and 13 (OR: 1.242, 95% CI: 1.042-1.480, P= 0.016) positively and factors 8 (OR:0.718, 95% CI: 0.651-0.793, P<0.001) and 11 (OR:0.862, 95% CI: 0.778-0.955, P= 0.004) were negatively associated with MetS.

**Table 4:** The unadjusted and adjusted odds ratio (OR) for the association between metabolites with VIP score values above 1.0 and metabolic syndrome (MetS).

	Crude model			Adjusted for age and sex		
Factors	OR	95% CI	P-value	OR	95% CI	P-value
1	1.002	(0.983-1.021)	0.866	1.004	(0.984-1.025)	0.686
2	1.000	(0.967-1.034)	0.993	1.010	(0.976-1.045)	0.583
3	1.103	(1.067-1.141)	**0.000**	1.165	(1.121-1.210)	**0.000**
4	0.945	(0.905-0.986)	**0.010**	0.959	(0.918-1.002)	0.059
5	1.006	(0.961-1.053)	0.806	1.035	(0.986-1.086)	0.166
6	1.076	(1.004-1.154)	**0.039**	1.058	(0.984-1.136)	0.126
7	1.201	(1.103-1.306)	**0.000**	1.257	(1.150-1.374)	**0.000**
8	0.757	(0.688-0.833)	**0.000**	0.718	(0.651-0.793)	**0.000**
9	1.750	(1.562-1.960)	**0.000**	1.883	(1.669-2.124)	**0.000**
10	1.065	(0.976-1.162)	0.158	1.132	(1.032-1.242)	**0.009**
11	0.832	(0.756-0.915)	**0.000**	0.862	(0.778-0.955)	**0.004**
12	1.009	(0.859-1.186)	0.911	1.070	(0.907-1.263)	0.422
13	1.230	(1.042-1.451)	**0.014**	1.242	(1.042-1.480)	**0.016**

Results were shown as odds ratio (OR) and the corresponding 95% confidence intervals (CI).

OD, odds ratio; CI, confidence interval.

Bold values denote statistical significance at the p < 0.05 level.

## Discussion

Recently, metabolomics studies have opened new insights into the identification of biomarkers for the diagnosis, monitoring, and risk prediction of metabolic disorders (7, 20, 27, 28). However, available evidence on the alteration of metabolites especially amino acids, and acylcarnitines in the context of MetS is little and conflicting (13, 20, 29), which warrants further exploration.

As revealed in the current study, two distinct amino acid patterns albeit with an opposing direction are significantly associated with MetS. The first one comprises elevated BCAAs (Val, Leu), aromatic amino acids (Phe, Tyr), Pro, Ala, Glu, Trp, Met, and the ratio of Asp to Asn, while the second one consists of decreased circulating levels of Gly, Ser, His, Asn, and citrulline.

Each of the two distinct amino acid patterns is mostly comprised of chemically and functionally correlated metabolites. The first pattern is composed of amino acids that their high levels are linked to impaired energy metabolism, dysregulation of insulin signaling, and the development of adverse cardiometabolic outcomes. To support this concept, data from *in vitro* studies and animal models revealed that Leu acts as a potential nutritional signal and shares metabolic regulatory function with insulin. Specifically, studies showed that leucine infusion inhibits insulin-stimulated glucose uptake, leading to desensitization of insulin signaling. Moreover, amino acids such as Ala, Val, or glutamine can augment glucose production and consequently induce hyperglycemia. Val also increased the free fatty acid uptake in serum, leading to the accumulation of neutral lipids and ATP production in the early stages of liver regeneration following resection (14, 15, 30). Additionally, dietary supplementation with aromatic amino acids has the potential to ameliorate hepatic steatosis by stimulating bile acid synthesis in mice (31). Accumulating evidence showed that methionine restriction has favorable effects on health since it leads to improvement of insulin signaling, elevated energy expenditure, and decreased oxidative damage and inflammation in the context of metabolic disorders. Moreover, hyperprolinemia is linked to impaired insulin secretion and dysregulated glucose homeostasis (32–34).

The second pattern is composed of amino acids that their decline is involved in the pathogenesis of metabolic disorders, particularly glucose intolerance, insulin resistance, and T2DM, albeit with conflicting findings. For instance, metabolic benefits mediated by glycine and histidine include the inhibition of oxidative stress and inflammation, and an inhibitory effect on gluconeogenesis and food intake Glycine also exerts positive effects on mitochondrial activity, detoxification processes, and regulation of hormones involved in glucose homeostasis (35, 36).

In agreement with the above-mentioned information, plasma levels of Leu, Val, and Phe have a moderate but significant correlation with FPG and TG levels; both are highly linked to the development of adverse cardiometabolic outcomes.

Although the current study cannot address the underlying mechanism linking altered amino acid profile to MetS components, our findings regarding the positive correlation of Leu, Val, and Phe with hypertriglyceridemia and hyperglycemia can be attributed to the functions of branched-chain amino acids as biological regulators of lipid and glucose metabolism and insulin signaling (14, 15).

In line with our data, *Sun et al.* showed increased levels of BCAAs, aromatic amino acids, Pro, Ala, Met, and Glu were linked to an elevated risk of MetS and its components in a Chinese Han population. They showed that Leu, Val, and Phe were positively associated with the concentration of TG and 2-h postprandial glucose (29). Several cross-sectional and prospective cohort studies also point toward the strong association between plasma levels of BCAA and aromatic amino acids and central obesity, T2D, and insulin resistance (11, 18, 37–39).

Okekunle et al. found a positive correlation between TG concentration and Leu, Val, and Phe in patients with obesity, T2D, and MetS. Compared to healthy individuals, a similar pattern was found in patients with multiple metabolic disorders; Val, Glu, Pro, and Ile were concomitantly elevated, while Gly was significantly decreased in multiple metabolic disorders (12).

An increased concentration of amino acids related to glutamate, alanine, and aromatic amino acid metabolism, but a lower level of glycine-serine-threonine metabolism-related amino acids in patients with Mets following a lifestyle modification program are other examples supporting altered amino acid profile in the context of MetS (40).

As for circulating levels of free carnitine and acylcarnitines, we found that only one distinct pattern including elevated free carnitine (C0), short-chain acylcarnitines (C3, C4DC, C5), and long-chain acylcarnitines (C16, C18OH) is associated with MetS. Among the above-mentioned acylcarnitines, C4DC was significantly associated with FPG levels.

In parallel, Libert et al. assigned adults (n=90) to a spectrum of metabolic wellness groups based on BMI and ATP III criteria for MetS: (i) lean metabolically well; (ii) obese metabolically well; (iii) obese metabolically unwell; and (iv) obese metabolically unwell with T2D. The results showed that patients who completely meet the ATP III criteria for MetS showed a higher level of both C3 carnitine and the ratio of C3 and C5 to total acylcarnitines in comparison with ones with a metabolically healthy status (20).

C3 acylcarnitine is formed from Val and Ile after interaction with branched-chain α-keto acid dehydrogenase (BCKD). However, C5 acylcarnitine can be produced from the breakdown of Iso and Leu before metabolism by BCKD (21). Hence, C5 and C3 acylcarnitine levels may reflect BCKD activity in the context of MetS. Specifically, the possible impairment of BCKD in MetS in a mechanism dependent on pathway rerouting can lead to the elevation of C5 concentration, while it can reduce C3 levels through upstream suppression (4). However, Libert’s study suggests that acylcarnitine can be formed both upstream and downstream of BCKD (20). In another study, Bene et al. observed higher levels of C3 and C4 acylcarnitines but lower levels of most of the medium-chain and long-chain acylcarnitine levels in patients with MetS and the patients with diabetes mellitus as compared to controls (7). It is well-established that BCAAs are generally increased in MetS, hence, it is tempting to speculate that an increased level of C3 acylcarnitine observed here is linked to elevated BCAA levels in the patients with Mets (4). However, the role of other pathways leading to C3 acylcarnitine formation cannot be ignored here.

Wolf et al. showed an elevated level of medium-chain acylcarnitines in individuals with high fasting respiratory quotient (RQ) as a risk factor for metabolic syndrome (41). RQ reflects the mix of fat and carbohydrates being oxidized (42). Hence, our data regarding an elevated level of medium-chain acylcarnitines indicates a reduced fatty acid oxidation capacity and a higher rate of incomplete fatty acid oxidation in patients with MetS.

Free carnitine facilitates the transport of activated fatty acids across the mitochondrial membrane and delivers them for β-oxidation to produce energy. In addition, it enhances acetyl and acyl group efflux out of the mitochondria into the cytosol as acylcarnitines. In line with our data, others reported higher free carnitine in patients with newly diagnosed T2D patients and individuals with obesity in comparison with controls (43, 44).

The finding of higher free carnitine in MetS is not unexpected and several possibilities derived from the pieces of literature can justify this finding. First, insulin resistance and metabolic disorders such as obesity and T2D are characterized by incomplete fatty acid oxidation that elevates acylcarnitine concentrations (44). Hence, it can be speculated that an increased concentration of free carnitine in the context of MetS is an endogenous effort to facilitate the transesterification of activated long-chain acyl-CoA to its acylcarnitine before entry into mitochondria. Another explanation may lie in the fact that free carnitine can induce oxidation of the branched-chain amino acids in several tissues and regulate ketogenesis by interaction with branched-chain acyl-CoA esters and pyruvate (45). Due to the increased plasma level of BCAA in our study, we can speculate that higher levels of free carnitine in patients with MetS might be a mechanism that facilitates BCAA oxidation in MetS.

However, several studies reported inconsistent data. For instance, Bene et al. reported a significantly lower level of free carnitine in T1D patients while no differences were found in the free carnitine concentrations between T2D and MetS patients and the controls (7). In another study, there was a trend of free carnitine levels being lower in patients with already-diagnosed diabetes mellitus as compared with controls (20).

This discrepancy stems from the complicated nature of the pathogenesis of metabolic disorders (1) as well as different degrees of involvement of impaired mitochondrial function and incomplete long-chain fatty acid oxidation pathways in the MetS (43).

According to sex differences, the plasma level of C0, some short-chain acylcarnitines, medium-chain acylcarnitines, and long-chain acylcarnitines were significantly higher in men in comparison with those in women in both study groups. As for amino acids, nearly all amino acids were significantly higher in men in comparison to those in women in both groups. These findings were in line with several studies conducted on other metabolic disorders (21, 46, 47). Specifically, it has been suggested that amino acids especially BCAA may be regulated differently by sex. Moreover, a higher concentration of some metabolites in men rather than in women can be attributed to larger muscle mass in men. However, the important role of sex steroids and their precursors in modulating carnitine turnover cannot be ignored when we interpret data on acylcarnitine levels (45, 48–50). Along with other studies, the aforementioned data can strengthen this concept that there is a sex-specific difference in the association between metabolites and MetS. However, more studies are needed to confirm this issue.

The main strength of our study is that metabolomics analysis was conducted on a large number of study participants who were randomly selected from a multi-regional cohort in Iran.

Another strength worth pointing out is that all anthropometric data and biochemical parameters were measured using standardized methods and rigorous statistical methodology was applied for data analysis. This ensures adequate statistical power for the generalization of the above findings at least in Iran.

To put these findings together, our data along with others can open new avenues for exploring potential biomarkers for clinical screening, diagnosis, and treatment. However, the present study has several limitations that merit consideration. First of all, changes in levels of several amino acids and acylcarnitines were found in patients with MetS in a study with a cross-sectional design which limits us to make conclusions about the contributory nature of metabolic changes in the progression of these conditions. Therefore, prospective cohort studies are warranted to replicate our findings in the future. More importantly, while we analyzed a large number of metabolites, the targeted nature of the metabolomics analysis mainly hinders the discovery of analytes that may be of importance for risk assessment while those were not analyzed.

In conclusion, our study showed that elevated plasma levels of some acylcarnitine and amino acid metabolites including Tyr, Leu, Val, Met, Trp, Phe, Thr, C0, C3, C4, Gly, Ser, Ala, Pro, C4DC, Glu, Asp, C18:2OH, citrulline, ornithine, C18OH, C18:1 OH were associated with MetS risk in Iranian adults with MetS. The present study further strengthens the existence of various mechanisms being responsible for MetS derangements. However, additional population cohorts should be undertaken to replicate our conclusion.

## Data availability statement

The original contributions presented in the study are included in the article/**Supplementary Material**. Further inquiries can be directed to the corresponding author.

## Ethics statement

Ethical approval for the current study was obtained from the ethics committee of the Endocrine and metabolism research institute (IR.TUMS.EMRI.REC. 1395.00141). The patients/participants provided their written informed consent to participate in this study.

## Author contributions

BL, FR, SE, and MK contributed to the study conception and design. BA EG, and HT provided study patients and monitored data and specimen collection. NN, AD-M, and SH performed the experiments. RG-G, SE, and SH analyzed the data. HT and HD wrote the manuscript. SE, MA, and NR edited the manuscript. All authors contributed to the article and approved the submitted version.

## Supplementary material

Supplementary Figure 1 Scree plot for principal component analysis (PCA) (IBM SPSS Statistics software version 26).

Supplementary Table 1 Binary logistic regression of 50 metabolites; 20 amino acids and 30 acylcarnitines for determining the predictive value of metabolites for metabolic syndrome. Results were shown as odds ratio (OR) and the corresponding 95% confidence intervals (CI). OD, odd ratio; FDR, false discovery rate; CI, confidence interval.

Supplementary Table 2 The univariate analysis of 50 metabolites; 20 amino acids and 30 acylcarnitines between the MetS group and non-MetS group. Concentrations are reported as mean± standard error of the mean (SEM). Significantly altered metabolites among groups’ classification using independent sample T-test or Mann-Whitney U test are marked in bold. FDR, false discovery rate.

Supplementary Table 3 The univariate analysis of 50 metabolites; 20 amino acids and 30 acylcarnitines among two study groups according to sex differences. Concentrations are reported as mean± standard error of the mean (SEM). Significantly altered metabolites were determined using an independent sample T-test or Mann-Whitney U test and marked in bold. FDR, false discovery rate.

Supplementary Table 4 Total variance explanation. Kaiser-Meyer-Olkin Measure of Sampling Adequacy: 0.858. Bartlett’s Test of Sphericity was significant (P-value < 0.001). Extraction Method: Principal Component Analysis. Rotation Method: Varimax with Kaiser Normalization.
