# Supplementary material for: The association between acylcarnitine and amino acids profile and metabolic syndrome and its components in Iranian adults: Data from STEPs 2016
Source: Front Endocrinol (Lausanne). 2023 Feb 27;14:1058952. doi: 10.3389/fendo.2023.1058952 (PMC10008865; doi:10.3389/fendo.2023.1058952)

**Supplementary Table 1.** Binary logistic regression of 50 metabolites; 20 amino acids and 30 acylcarnitines for determining the predictive value of metabolites for metabolic syndrome.

| Metabolites  (µmol/L) |  | Crude model |  | Adjusted by age and sex | | |
| --- | --- | --- | --- | --- | --- | --- |
|  | OR | 95% CI | FDR | OR | 95% CI | FDR |
| C0 | 1.263 | (1.124-1.420) | **0.000** | 1.312 | (1.163-1.481) | **0.000** |
| C2 | 1.129 | (1.005-1.267) | 0.102 | 1.094 | (0.972-1.232) | 0.291 |
| C3 | 1.343 | (1.189-1.517) | **0.000** | 1.484 | (1.300-1.693) | **0.000** |
| C3DC | 0.99 | (0.883-1.111) | 0.971 | 1.036 | (0.920-1.166) | 0.657 |
| C4 | 0.993 | (0.885-1.113) | 0.971 | 0.995 | (0.885-1.118) | 0.954 |
| C4OH | 1.065 | (0.95-1.194) | 0.431 | 1.054 | (0.938-1.185) | 0.592 |
| C4DC | 1.499 | (1.301-1.727) | **0.000** | 1.574 | (1.354-1.830) | **0.000** |
| C5 | 1.055 | (0.941-1.182) | 0.489 | 1.177 | (1.042-1.330) | **0.025** |
| C5:1 | 1.062 | (0.947-1.19) | 0.451 | 1.066 | (0.948-1.199) | 0.492 |
| C5OH | 1.023 | (0.913-1.147) | 0.839 | 1.080 | (0.958-1.217) | 0.369 |
| C5DC | 0.877 | (0.78-0.985) | 0.068 | 0.964 | (0.850-1.093) | 0.657 |
| C6 | 0.99 | (0.882-1.111) | 0.971 | 1.005 | (0.894-1.129) | 0.954 |
| C8 | 1.006 | (0.897-1.127) | 0.971 | 1.039 | (0.925-1.166) | 0.657 |
| C8:1 | 1.118 | (0.997-1.254) | 0.129 | 1.083 | (0.964-1.217) | 0.343 |
| C10 | 1 | (0.892-1.121) | 0.998 | 1.035 | (0.921-1.163) | 0.657 |
| C10:1 | 1.001 | (0.893-1.122) | 0.998 | 1.043 | (0.928-1.171) | 0.657 |
| C12 | 0.907 | (0.806-1.021) | 0.232 | 0.939 | (0.834-1.058) | 0.512 |
| C14 | 0.932 | (0.828-1.05) | 0.418 | 0.945 | (0.837-1.065) | 0.574 |
| C14:1 | 0.914 | (0.813-1.027) | 0.262 | 0.916 | (0.813-1.032) | 0.298 |
| C14:2 | 0.923 | (0.822-1.037) | 0.321 | 0.967 | (0.859-1.089) | 0.657 |
| C14OH | 1.081 | (0.963-1.213) | 0.321 | 1.083 | (0.958-1.223) | 0.369 |
| C16 | 1.139 | (1.016-1.278) | 0.068 | 1.172 | (1.039-1.320) | **0.025** |
| C16OH | 1.089 | (0.971-1.222) | 0.279 | 1.112 | (0.984-1.256) | 0.199 |
| C16:1OH | 0.958 | (0.852-1.077) | 0.609 | 0.952 | (0.844-1.075) | 0.620 |
| C16:1 | 1.005 | (0.896-1.126) | 0.971 | 0.954 | (0.846-1.076) | 0.625 |
| C18 | 0.942 | (0.838-1.058) | 0.451 | 0.984 | (0.872-1.109) | 0.852 |
| C18:1 | 1.021 | (0.911-1.145) | 0.846 | 1.014 | (0.901-1.142) | 0.862 |
| C18OH | 1.234 | (1.087-1.401) | **0.003** | 1.248 | (1.090-1.428) | **0.003** |
| C18:1OH | 1.071 | (0.902-1.272) | 0.581 | 1.051 | (0.885-1.248) | 0.657 |
| C18:2OH | 1.043 | (0.928-1.171) | 0.609 | 1.051 | (0.931-1.186) | 0.620 |
| Alanine | 2.089 | (1.823-2.393) | **0.000** | 2.107 | (1.834-2.420) | **0.000** |
| Aspartic Acid | 0.944 | (0.841-1.058) | 0.453 | 1.001 | (0.890-1.127) | 0.980 |
| Glutamic Acid | 1.184 | (1.048-1.338) | **0.021** | 1.315 | (1.152-1.502) | **0.000** |
| Leucine | 1.542 | (1.362-1.746) | **0.000** | 2.052 | (1.769-2.381) | **0.000** |
| Methionine | 1.035 | (0.924-1.161) | 0.681 | 1.164 | (1.030-1.315) | **0.039** |
| Phenylalanine | 1.283 | (1.134-1.451) | **0.000** | 1.408 | (1.230-1.611) | **0.000** |
| Tyrosine | 1.525 | (1.349-1.723) | **0.000** | 1.646 | (1.448-1.871) | **0.000** |
| Valine | 1.774 | (1.556-2.023) | **0.000** | 2.148 | (1.855-2.487) | **0.000** |
| Arginine | 1.007 | (0.898-1.128) | 0.971 | 1.049 | (0.933-1.180) | 0.620 |
| Citrulline | 0.747 | (0.662-0.842) | **0.000** | 0.772 | (0.679-0.879) | **0.000** |
| Glycine | 0.792 | (0.704-0.892) | **0.000** | 0.721 | (0.637-0.817) | **0.000** |
| Ornithine | 0.924 | (0.824-1.037) | 0.321 | 0.963 | (0.854-1.085) | 0.657 |
| Proline | 1.254 | (1.115-1.410) | **0.000** | 1.378 | (1.214-1.564) | **0.000** |
| Threonine | 0.874 | (0.778-0.981) | 0.066 | 0.914 | (0.812-1.030) | 0.291 |
| Serine | 0.671 | (0.592-0.761) | **0.000** | 0.663 | (0.583-0.753) | **0.000** |
| Histidine | 0.849 | (0.756-0.953) | **0.020** | 0.874 | (0.776-0.984) | 0.064 |
| Lysine | 0.938 | (0.836-1.052) | 0.427 | 0.965 | (0.859-1.086) | 0.657 |
| Tryptophane | 1.125 | (1.003-1.262) | 0.104 | 1.262 | (1.115-1.428) | **0.000** |
| Asparagine | 0.762 | (0.675-0.860) | **0.000** | 0.795 | (0.703-0.899) | **0.000** |
| Glutamine | 0.937 | (0.835-1.05) | 0.427 | 0.969 | (0.861-1.090) | 0.663 |
| Glu/Gln | 1.100 | (0.972-1.244) | 0.262 | 1.132 | (0.998-1.285) | 0.128 |
| Asp/Asn | 1.222 | (1.083-1.380) | **0.003** | 1.218 | (1.076-1.379) | **0.006** |

Results were shown as odds ratio (OR) and the corresponding 95% confidence intervals (CI).

OD, odd ratio; FDR, false discovery rate; CI, confidence interval

**Supplementary Table 2**. The univariate analysis of 50 metabolites; 20 amino acids and 30 acylcarnitines between the MetS group and non-MetS group

| Metabolites (µmol/L) | Non-metabolic syndrome | | | Metabolic syndrome | | | FOR |
| --- | --- | --- | --- | --- | --- | --- | --- |
| C0 | 55.121 | ± | 0.492 | 58.121 | ± | 0.577 | **0.000** |
| C2 | 14.323 | ± | 0.185 | 14.872 | ± | 0.187 | **0.021** |
| C3 | 0.832 | ± | 0.013 | 0.940 | ± | 0.018 | **0.000** |
| C3DC | 0.088 | ± | 0.002 | 0.087 | ± | 0.002 | 0.746 |
| C4 | 0.514 | ± | 0.021 | 0.510 | ± | 0.019 | **0.000** |
| C4OH | 0.057 | ± | 0.001 | 0.058 | ± | 0.001 | 0.063 |
| C4DC | 0.067 | ± | 0.001 | 0.080 | ± | 0.002 | **0.000** |
| C5 | 0.232 | ± | 0.004 | 0.237 | ± | 0.004 | 0.183 |
| C5:1 | 0.045 | ± | 0.001 | 0.047 | ± | 0.001 | 0.874 |
| C5OH | 0.067 | ± | 0.001 | 0.067 | ± | 0.001 | 0.703 |
| C5DC | 0.335 | ± | 0.005 | 0.320 | ± | 0.005 | 0.170 |
| C6 | 0.216 | ± | 0.009 | 0.214 | ± | 0.009 | 0.349 |
| C8 | 0.333 | ± | 0.014 | 0.335 | ± | 0.014 | 0.553 |
| C8:1 | 0.328 | ± | 0.007 | 0.348 | ± | 0.008 | 0.170 |
| C10 | 0.432 | ± | 0.016 | 0.432 | ± | 0.017 | 0.535 |
| C10:1 | 0.395 | ± | 0.014 | 0.395 | ± | 0.013 | 0.596 |
| C12 | 0.153 | ± | 0.004 | 0.146 | ± | 0.003 | 0.799 |
| C14 | 0.060 | ± | 0.001 | 0.058 | ± | 0.001 | 0.958 |
| C14:1 | 0.137 | ± | 0.003 | 0.131 | ± | 0.003 | 0.817 |
| C14:2 | 0.101 | ± | 0.002 | 0.097 | ± | 0.002 | 0.546 |
| C14OH | 0.013 | ± | 0.000 | 0.013 | ± | 0.000 | 0.238 |
| C16 | 0.182 | ± | 0.002 | 0.190 | ± | 0.002 | **0.005** |
| C16OH | 0.011 | ± | 0.000 | 0.012 | ± | 0.000 | 0.380 |
| C16:1OH | 0.019 | ± | 0.000 | 0.018 | ± | 0.000 | 0.890 |
| C16:1 | 0.049 | ± | 0.001 | 0.049 | ± | 0.001 | 0.328 |
| C18 | 0.068 | ± | 0.001 | 0.067 | ± | 0.001 | 0.553 |
| C18:1 | 0.185 | ± | 0.003 | 0.187 | ± | 0.003 | 0.559 |
| C18OH | 0.009 | ± | 0.000 | 0.010 | ± | 0.000 | **0.003** |
| C18:1OH | 0.013 | ± | 0.000 | 0.014 | ± | 0.002 | 0.817 |
| C18:2OH | 0.032 | ± | 0.001 | 0.033 | ± | 0.001 | 0.380 |
| Alanine | 388.21 | ± | 3.51 | 452.15 | ± | 4.13 | **0.000** |
| Aspartic Acid | 12.83 | ± | 0.15 | 12.61 | ± | 0.16 | 0.535 |
| Glutamic Acid | 66.88 | ± | 0.61 | 69.27 | ± | 0.58 | **0.003** |
| Leucine | 120.67 | ± | 1.01 | 131.71 | ± | 1.17 | **0.000** |
| Methionine | 28.29 | ± | 0.23 | 28.50 | ± | 0.26 | 0.746 |
| Phenylalanine | 63.26 | ± | 0.45 | 66.37 | ± | 0.64 | **0.003** |
| Tyrosine | 68.81 | ± | 0.54 | 74.75 | ± | 0.65 | **0.000** |
| Valine | 249.83 | ± | 1.91 | 277.45 | ± | 2.32 | **0.000** |
| Arginine | 69.57 | ± | 0.75 | 69.70 | ± | 0.87 | 0.755 |
| Citrulline | 39.85 | ± | 0.41 | 36.93 | ± | 0.44 | **0.000** |
| Glycine | 275.29 | ± | 3.17 | 257.26 | ± | 3.34 | **0.000** |
| Ornithine | 91.99 | ± | 0.95 | 90.14 | ± | 0.99 | **0.319** |
| Proline | 246.05 | ± | 3.21 | 264.77 | ± | 3.66 | **0.000** |
| Threonine | 140.17 | ± | 1.42 | 135.47 | ± | 1.46 | 0.063 |
| Serine | 107.74 | ± | 1.14 | 97.00 | ± | 1.20 | **0.000** |
| Histidine | 84.65 | ± | 0.63 | 81.90 | ± | 0.78 | **0.035** |
| Lysine | 181.48 | ± | 1.76 | 178.62 | ± | 1.90 | 0.553 |
| Tryptophane | 69.65 | ± | 0.64 | 71.68 | ± | 0.79 | 0.093 |
| Asparagine | 49.54 | ± | 0.78 | 44.49 | ± | 0.79 | **0.000** |
| Glutamine | 522.94 | ± | 4.95 | 514.80 | ± | 5.26 | 0.553 |
|  |  |  |  |  |  |  |  |

Concentrations are reported as mean± standard error of the mean (SEM). Significantly altered metabolites among groups’ classification using independent sample T-test or Mann-Whitney U test are marked in bold. FDR, false discovery rate.

**Supplementary Table 3.** The univariate analysis of 50 metabolites; 20 amino acids and 30 acylcarnitines among two study groups according to sex differences.

|  | **Non-metabolic syndrome** | | | | | **Metabolic syndrome** | | | | |
| --- | --- | --- | --- | --- | --- | --- | --- | --- | --- | --- |
| **Metabolites** | **Female** | | **Male** | | **FDR** | **Female** | | **Male** | | **FDR** |
| (µmol/L) | mean | SEM | mean | SEM |  | mean | SEM | mean | SEM |  |
| C0 | 53.378 | 0.723 | 56.519 | 0.663 | **0.002** | 56.681 | 0.706 | 60.452 | 0.970 | **0.006** |
| C2 | 14.579 | 0.288 | 14.118 | 0.239 | 0.076 | 14.970 | 0.242 | 14.713 | 0.294 | 0.551 |
| C3 | 0.741 | 0.016 | 0.905 | 0.019 | **0.000** | 0.869 | 0.021 | 1.054 | 0.031 | **0.000** |
| C3DC | 0.081 | 0.003 | 0.093 | 0.002 | **0.000** | 0.081 | 0.002 | 0.098 | 0.003 | **0.000** |
| C4 | 0.512 | 0.034 | 0.515 | 0.027 | 0.190 | 0.503 | 0.023 | 0.522 | 0.033 | 0.213 |
| C4OH | 0.056 | 0.001 | 0.058 | 0.002 | 0.790 | 0.059 | 0.001 | 0.058 | 0.002 | 0.954 |
| C4DC | 0.063 | 0.001 | 0.071 | 0.001 | **0.000** | 0.077 | 0.002 | 0.085 | 0.003 | **0.014** |
| C5 | 0.200 | 0.005 | 0.257 | 0.006 | **0.000** | 0.215 | 0.005 | 0.273 | 0.007 | **0.000** |
| C5:1 | 0.045 | 0.002 | 0.044 | 0.001 | 0.782 | 0.044 | 0.002 | 0.050 | 0.003 | 0.093 |
| C5OH | 0.063 | 0.001 | 0.070 | 0.001 | **0.000** | 0.064 | 0.001 | 0.072 | 0.002 | **0.000** |
| C5DC | 0.293 | 0.006 | 0.369 | 0.007 | **0.000** | 0.289 | 0.005 | 0.370 | 0.008 | **0.000** |
| C6 | 0.205 | 0.015 | 0.225 | 0.011 | 0.457 | 0.206 | 0.007 | 0.226 | 0.020 | 0.244 |
| C8 | 0.292 | 0.014 | 0.365 | 0.022 | **0.050** | 0.314 | 0.014 | 0.368 | 0.030 | **0.007** |
| C8:1 | 0.338 | 0.010 | 0.320 | 0.008 | 0.302 | 0.359 | 0.011 | 0.331 | 0.013 | 0.183 |
| C10 | 0.380 | 0.018 | 0.473 | 0.024 | **0.009** | 0.407 | 0.018 | 0.472 | 0.033 | **0.011** |
| C10:1 | 0.349 | 0.012 | 0.432 | 0.023 | **0.028** | 0.369 | 0.013 | 0.439 | 0.027 | **0.002** |
| C12 | 0.142 | 0.006 | 0.163 | 0.005 | **0.000** | 0.137 | 0.004 | 0.160 | 0.005 | **0.000** |
| C14 | 0.057 | 0.002 | 0.062 | 0.001 | **0.000** | 0.055 | 0.001 | 0.062 | 0.002 | **0.000** |
| C14:1 | 0.132 | 0.005 | 0.141 | 0.004 | 0.161 | 0.128 | 0.004 | 0.134 | 0.004 | 0.227 |
| C14:2 | 0.094 | 0.003 | 0.108 | 0.003 | **0.004** | 0.091 | 0.002 | 0.107 | 0.003 | **0.000** |
| C14OH | 0.012 | 0.000 | 0.013 | 0.000 | **0.006** | 0.013 | 0.000 | 0.014 | 0.000 | **0.037** |
| C16 | 0.175 | 0.003 | 0.188 | 0.003 | **0.009** | 0.185 | 0.003 | 0.198 | 0.004 | **0.007** |
| C16OH | 0.011 | 0.000 | 0.012 | 0.000 | **0.000** | 0.011 | 0.000 | 0.012 | 0.000 | **0.004** |
| C16:1OH | 0.018 | 0.001 | 0.019 | 0.000 | **0.006** | 0.018 | 0.000 | 0.019 | 0.001 | 0.113 |
| C16:1 | 0.050 | 0.002 | 0.049 | 0.001 | 0.194 | 0.051 | 0.001 | 0.046 | 0.001 | **0.025** |
| C18 | 0.064 | 0.002 | 0.072 | 0.001 | **0.000** | 0.063 | 0.001 | 0.073 | 0.002 | **0.000** |
| C18:1 | 0.181 | 0.004 | 0.189 | 0.004 | 0.302 | 0.186 | 0.004 | 0.188 | 0.005 | 0.909 |
| C18OH | 0.008 | 0.000 | 0.009 | 0.000 | **0.000** | 0.009 | 0.000 | 0.010 | 0.000 | 0.111 |
| C18:1OH | 0.013 | 0.000 | 0.013 | 0.000 | 0.100 | 0.015 | 0.003 | 0.013 | 0.000 | 0.207 |
| C18:2OH | 0.032 | 0.002 | 0.032 | 0.001 | 0.058 | 0.032 | 0.001 | 0.035 | 0.002 | 0.193 |
| Alanine | 382.075 | 5.102 | 393.121 | 4.810 | 0.128 | 452.979 | 5.405 | 450.808 | 6.393 | 0.811 |
| Aspartic Acid | 12.492 | 0.217 | 13.100 | 0.196 | **0.041** | 12.012 | 0.201 | 13.585 | 0.261 | **0.000** |
| Glutamic Acid | 63.308 | 0.769 | 69.746 | 0.888 | **0.000** | 66.581 | 0.712 | 73.633 | 0.930 | **0.000** |
| Leucine | 109.966 | 1.234 | 129.245 | 1.366 | **0.000** | 123.226 | 1.327 | 145.444 | 1.805 | **0.000** |
| Methionine | 26.830 | 0.328 | 29.462 | 0.313 | **0.000** | 27.181 | 0.305 | 30.635 | 0.436 | **0.000** |
| Phenylalanine | 60.560 | 0.633 | 65.422 | 0.610 | **0.000** | 64.389 | 0.902 | 69.574 | 0.793 | **0.000** |
| Tyrosine | 66.739 | 0.786 | 70.478 | 0.728 | **0.004** | 72.506 | 0.826 | 78.391 | 1.017 | **0.000** |
| Valine | 234.386 | 2.437 | 262.208 | 2.676 | **0.000** | 266.518 | 2.871 | 295.141 | 3.590 | **0.000** |
| Arginine | 68.594 | 1.096 | 70.351 | 1.027 | 0.185 | 67.464 | 1.065 | 73.309 | 1.445 | **0.000** |
| Citrulline | 36.778 | 0.605 | 42.305 | 0.517 | **0.000** | 34.833 | 0.524 | 40.325 | 0.705 | **0.000** |
| Glycine | 292.938 | 5.460 | 261.152 | 3.516 | **0.000** | 272.426 | 4.737 | 232.698 | 3.570 | **0.000** |
| Ornithine | 85.779 | 1.258 | 96.972 | 1.338 | **0.000** | 88.958 | 1.274 | 92.060 | 1.544 | 0.076 |
| Proline | 224.612 | 4.715 | 263.232 | 4.178 | **0.000** | 252.645 | 4.633 | 284.403 | 5.740 | **0.000** |
| Threonine | 135.930 | 2.133 | 143.570 | 1.891 | **0.009** | 133.995 | 1.885 | 137.845 | 2.310 | 0.168 |
| Serine | 110.389 | 1.821 | 105.616 | 1.439 | 0.115 | 97.521 | 1.534 | 96.165 | 1.912 | 0.454 |
| Histidine | 84.422 | 0.923 | 84.839 | 0.857 | 0.683 | 80.873 | 1.009 | 83.564 | 1.206 | 0.213 |
| Lysine | 176.925 | 2.406 | 185.140 | 2.512 | 0.053 | 175.757 | 2.339 | 183.254 | 3.198 | 0.141 |
| Tryptophane | 65.984 | 0.840 | 72.592 | 0.899 | **0.000** | 68.927 | 0.992 | 76.139 | 1.250 | **0.000** |
| Asparagine | 47.613 | 1.098 | 51.088 | 1.100 | **0.023** | 42.478 | 0.994 | 47.746 | 1.269 | **0.000** |
| Glutamine | 507.707 | 6.969 | 535.148 | 6.891 | **0.012** | 504.274 | 6.448 | 531.844 | 8.898 | **0.044** |

Concentrations are reported as mean± standard error of the mean (SEM). Significantly altered metabolites were determined using an independent sample T-test or Mann-Whitney U test and marked in bold. FDR, false discovery rate.

**Supplementary Table 4.** Total variance explanation

| Factors | Eigenvalues | % Of Variance | Cumulative % |
| --- | --- | --- | --- |
| 1 | 11.12 | 22.24 | 22.24 |
| 2 | 6.52 | 13.04 | 35.28 |
| 3 | 3.50 | 7.00 | 42.28 |
| 4 | 3.16 | 6.32 | 48.60 |
| 5 | 2.08 | 4.17 | 52.77 |
| 6 | 1.66 | 3.31 | 56.08 |
| 7 | 1.59 | 3.18 | 59.26 |
| 8 | 1.53 | 3.06 | 62.32 |
| 9 | 1.27 | 2.53 | 64.85 |
| 10 | 1.19 | 2.37 | 67.22 |
| 11 | 1.06 | 2.13 | 69.35 |
| 12 | 1.02 | 2.03 | 71.38 |
| 13 | 1.00 | 2.00 | 73.38 |

Kaiser-Meyer-Olkin Measure of Sampling Adequacy: 0.858

Bartlett's Test of Sphericity was significant (P-value < 0.001)

Extraction Method: Principal Component Analysis.

Rotation Method: Varimax with Kaiser Normalization.

**Supplementary Figure 1.** Scree plot for principal component analysis (PCA) (IBM SPSS Statistics software version 26).


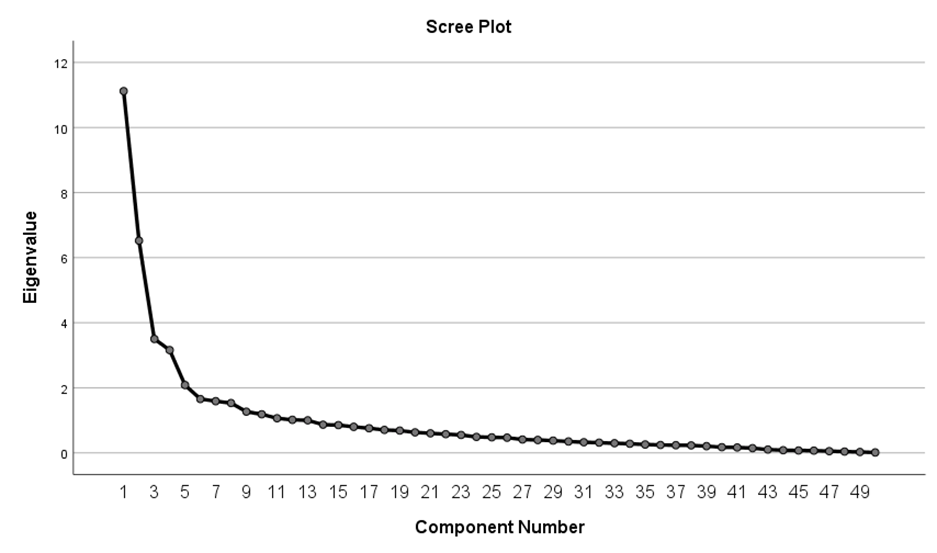

Supplement: Supplementary Figure 1 — Scree plot for principal component analysis (PCA) (IBM SPSS Statistics software version 26). [file DataSheet_1.docx]
